# Integrated New Approach Methodologies Reveal the Potential Role of 2,7-Dibromocarbazole in Parkinson’s Disease via Monoamine Oxidase B Inhibition and Dopaminergic Dysfunction

**DOI:** 10.1021/acs.est.5c14167

**Published:** 2026-02-23

**Authors:** Xinhe Lu, Yuhang Luo, Pei Peng, Xingyue Xing, Wei Xia, Hongyan Yin, Hanzeng Li, Shunqing Xu

**Affiliations:** 1 School of Environmental and Science and Engineering, 74629 Hainan University, Haikou 570228, China; 2 Key Laboratory of Environment and Health (HUST), Ministry of Education & Ministry of Environmental Protection, School of Public Health, Tongji Medical College, 540681 Huazhong University of Science and Technology, Wuhan, Hubei 430030, China; 3 School of Life and Health Sciences, 74629 Hainan University, Haikou 570228, China; 4 School of Tropical Agriculture and Forestry, 74629 Hainan University, Haikou 570228, China

**Keywords:** PHCZs, Parkinson’s disease, network toxicology, transcriptomic analysis, neurotoxicity

## Abstract

The neurotoxicity of emerging contaminants, polyhalogenated carbazoles (PHCZs), is elusive. In this study, we investigated the potential toxicity of 13 prevalent PHCZs utilizing a network toxicology approach, which revealed shared molecular targets associated with Parkinson’s disease (PD). Molecular docking simulations assessed the binding affinities of these PHCZs for eight key PD-related targets, identifying monoamine oxidase B (MAOB) as a critical target. Among the dihalogenated PHCZs, 2,7-dibromocarbazole (2,7-BCZ) exhibited the highest binding affinity to MAOB. Comparative molecular docking and dynamics simulations suggest that the inhibition of MAOB activity by 2,7-BCZ is a potential initiating event in PHCZ-induced neurological disorders. *In vivo* experiments confirmed that 2,7-BCZ exposure highly correlates with α-synuclein aggregation, a hallmark of PD pathology. Transcriptomic sequencing of 2,7-BCZ-exposed SH-SY5Y cells, combined with analysis of public PD microarray data, identified shared transcriptional alterations in genes including *CLSTN2*, *CBLN1*, *AGTR1*, *DLK1*, and *DDC*. By integrating pathways from PD-related targets of PHCZs, differentially expressed genes in 2,7-BCZ-exposed cells, and public PD data sets, we further elucidated key biological pathways through which 2,7-BCZ may contribute to PD pathogenesis, particularly dopaminergic synapse function and neurodevelopmental regulation. Collectively, this study not only highlights the potential role of PHCZs in PD, elucidating the potential biological mechanisms by which PHCZs may exacerbate PD, but also exemplifies an innovative, animal-sparing approach using New Approach Methodologies (NAMs) to assess environmental pollutants’ risks in neurodegenerative adverse outcomes.

## Graphical abstract

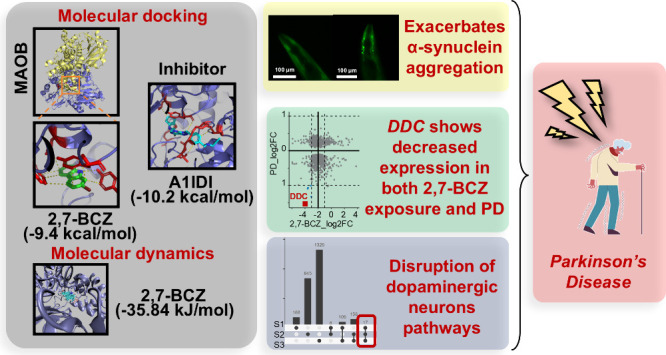

## 1 Introduction

Polyhalogenated carbazoles (PHCZs), a class of dioxin-like compounds (DLCs), have recently attracted significant attention due to their widespread environmental presence and potential toxicity.^1,2^ Recent reviews have further underscored the urgency of research on PHCZs and the imperative for their effective control.^3,4^ This urgency is heightened by their environmental persistence, widespread detection, and potential toxicological impacts. These persistent organic pollutants have been detected across diverse environmental matrices, including farmlands,^5^ freshwater and estuarine systems,^6^ indoor dust,^7^ earthworms,^8^ PM2.5 particles,^9^ Arctic soils,^10^ and human urine.^11^ In Chinese farmlands, 3,6-dichlorocarbazole (3,6-CCZ) is the predominant congener (40.67%) in soil, followed by 3-chlorocarbazole (3-CCZ) (14.51%).^5^ In Beijing’s atmospheric PM2.5, 3-CCZ, 3-bromocarbazole (3-BCZ), and 3,6-CCZ constitute 93% of PHCZs.^7^ Notably, PHCZ concentrations in electronic waste disposal site soils (896–41,362 ng kg^–1^) far exceed those of other pollutants.^12^ Industrial emissions, particularly from dyeing and textile processes, are major contributors to elevated environmental PHCZ levels in the Yangtze River Delta,^13^ with concentrations surpassing those of polychlorinated biphenyls and decabromodiphenyl ether.^14^ PHCZs exhibit bioaccumulation, biomagnification, and dioxin-like effects likely due to structural similarities.^15^ Their environmental persistence and toxicity, including liver metabolism disruption via gut microbiota alterations,^16^ underscore significant ecological and health risks.

Despite the growing evidence of PHCZs’ environmental prevalence and toxicity, their overall toxicological profiles and the underlying mechanisms, particularly in relation to neurodegenerative diseases such as Parkinson’s disease (PD), remain poorly understood. Studies have demonstrated that aryl hydrocarbon receptor (AhR) activation accounts for PHCZs’ dioxin-like effects.^17,18^ Moreover, developmental toxicity in zebrafish^19–21^ and their impacts on soil microbial processes^22,23^ have also been documented. However, specific pathways linking PHCZ exposure to neurotoxicity are unclear. Evidence has shown that 2,7-dibromocarbazole (2,7-BCZ) at 50 μg/L induces brain damage in animal models, characterized by multinucleated cells and nuclear pyknosis,^24^ yet the molecular mechanisms underlying these effects remain unknown. Furthermore, PHCZs’ bioaccumulation in fish brains,^25^ together with their roles in promoting reactive oxygen species (ROS) production, lipid peroxidation, and DNA damage,^26^ suggests potential neurodegenerative risks. While previous studies have primarily focused on the environmental occurrence and general toxicity of PHCZs,^17,20^ their potential role in neurodegenerative diseases, particularly PD, remains largely unknown. This study integrates computational, transcriptomic, and *in vivo* approaches to systematically evaluate the neurotoxicity of 2,7-BCZ and elucidate its underlying mechanisms, thereby filling a critical knowledge gap in PHCZ risk assessment. Nevertheless, few studies integrate multiomics approaches, such as transcriptomics, with computational toxicology and machine learning to map these pathways comprehensively. Current targeted analytical methods for PHCZ detection^16,27–29^ limit the identification of nontargeted halogenated compounds, potentially underestimating exposure risks. Moreover, the potential combined effects of PHCZs with other environmental contaminants warrant further investigation to better understand their neurotoxic risk profiles.^30–32^

In recent years, New Approach Methodologies (NAMs), with network toxicology as a key component, have emerged as powerful interdisciplinary tools for elucidating the multifactorial toxic mechanisms of chemicals. Network toxicology integrates diverse data sets across disciplines to facilitate the construction of more refined toxicity models, thereby revealing systemic mechanisms at the molecular, cellular, and organismal levels. Unlike traditional single-target approaches, it leverages bioinformatics and computational tools to analyze large-scale toxicological data, enabling the identification of potential toxic mechanisms and biomarkers. Moreover, it allows simulation of interactions between biological systems and chemical variants, providing predictive insights under diverse exposure conditions. Such capabilities are essential for the comprehensive assessment of chemical pollutants including PHCZs. We hypothesized that PHCZs may contribute to PD-like neurotoxicity by direct interaction with an unidentified molecular target that resides in dopaminergic neurons. To test this, we aim to (1) predict molecular targets using network toxicology and molecular docking, (2) characterize transcriptomic alterations in human neuronal cells, and (3) validate key neurotoxic phenotypes in a*C. elegans*model. To the best of our knowledge, this is the first study to systematically evaluate the neurotoxicity of PHCZs using an integrated NAMs framework, bridging computational prediction with experimental validation in the context of PD.

## 2 Materials and Methods

### 2.1 Toxicity Prediction Analysis

The SMILES molecular descriptors of PHCZs were retrieved from PubChem (https://www.pubchem.ncbi.nlm.nih.gov/). Toxicity predictions were conducted using the ADMETLab platform^33^ (https://admetlab3.scbdd.com/server/screening) by inputting the corresponding SMILES strings.

### 2.2 Collection of Toxic Targets of PHCZs and PD

The potential target genes of PHCZs were predicted using several online tools, including SuperPRED^34^ (https://prediction.charite.de/index.php), NetInfer^35^ (https://lmmd.ecust.edu.cn/netinfer/), and SEA^36^ (Similarity Ensemble Approach, https://sea.bkslab.org/). SuperPRED constructs compound–target interaction data derived from SuperTarget, ChEMBL, and BindingDB, with weakly binding interactions (*e.g.*, *K*_i_ or IC50 values > 10 μM) excluded. NetInfer utilizes a network-based inference algorithm to predict targets and associated therapeutic and adverse effects. SEA associates proteins based on the chemical similarity of their ligands. The SMILES representations of all PHCZs were input into the aforementioned tools, and the resulting targets were combined and deduplicated to obtain the final list of PHCZ-associated genes. PD-related target genes were retrieved from GeneCards^37^ (https://www.genecards.org/) and OMIM (https://www.omim.org/). In GeneCards, a search for “Parkinson” was conducted under the “Disorders” category, selecting only “Protein Coding” genes. In OMIM, a direct search for “Parkinson” was performed. The resulting genes from both databases were merged and deduplicated to generate the final set of PD-related genes.

### 2.3 Gene Enrichment Analysis

Gene ontology^38^ (GO) and Kyoto Encyclopedia of Genes and Genomes (KEGG) enrichment analyses were conducted using the R package “clusterProfiler” with a significance threshold of adjusted *P <* 0.05.^39,40^ For GO enrichment, the top three terms in each category, namely, biological process, cellular component, and molecular function, were selected based on gene count for visualization.

### 2.4 Protein–Protein Interaction (PPI) Network Analysis

The intersecting genes between PHCZs and PD target genes were subjected to protein–protein interaction (PPI) network analysis. These genes were uploaded to the STRING database (https://cn.string-db.org/) to retrieve protein interaction relationships.^41^ The network was visualized using Cytoscape (version 3.10.3).^42^

### 2.5 Molecular Docking

Protein structures were retrieved from the Protein Data Bank (PDB), and the 2,7-BCZ structure file was downloaded from the PubChem database. Molecular docking was performed using CBDock2 (https://cadd.labshare.cn/cb-dock2/php/index.php).^43^ The binding energy (kcal/mol) was calculated for the site with the highest binding affinity.

### 2.6 Molecular Dynamic Analysis

Molecular dynamics (MD) simulations of monoamine oxidase B (MAOB) in complex with 2,7-BCZ were performed using GROMACS (version 2025.4).^44–46^ Atomic charges for the small molecule were assigned using Multiwfn,^47,48^ and its topology file was generated based on the GAFF force field via sobtop.^49,50^ The protein topology was constructed using the AMBER99SB-ILDN force field, with the TIP3P model employed for water molecules.^51,52^ Following energy minimization, NVT equilibration, and NPT equilibration, molecular dynamics simulations were subsequently performed. The binding free energy was calculated using the gmx_MMPBSA method.^53^ For the visualization of results, the software tools QtGrace and VMD were employed.^54^

### 2.7 Quantitative MAOB assays

MAOB protein levels were quantified using a commercially available human MAOB enzyme-linked immunosorbent assay kit (EH2511, Wuhan Fine Biotech Co., Ltd.) according to the manufacturer’s instructions. Briefly, cell lysates or tissue homogenates were prepared and centrifuged, and the resulting supernatants were collected for analysis. Standards and samples were added to the antibody-precoated wells and incubated at 37 °C for 90 min. After the plates were washed, a biotin-conjugated detection antibody was added, followed by a streptavidin-HRP conjugate. The tetramethylbenzidine substrate was then used for color development, and the reaction was stopped with sulfuric acid. Absorbance was measured at 450 nm by using a microplate reader. Protein concentrations were determined by interpolating sample values against a standard curve generated from known MAOB concentrations.

### 2.8 Evaluation of the Effect of 2,7-BCZ on α-Synuclein Aggregation

The *C. elegans* strain NL5901 [*unc-*54*p*::α-synuclein::YFP] was used to investigate α-synuclein aggregation in body wall muscle cells. This strain expresses human α-synuclein fused to yellow fluorescent protein (YFP) under the control of the *unc-*54 promoter. NL5901 was obtained from the Caenorhabditis Genetics Center (CGC, University of Minnesota, USA).

Worms were maintained at 20 °C on nematode growth medium (NGM) plates seeded with *E. coli* OP50 as a food source, following standard protocols. Synchronized worms were generated by hypochlorite bleaching adults to obtain eggs, which were allowed to hatch overnight in M9 buffer, followed by transfer to fresh NGM plates. NL5901 worms were grown on NGM plates seeded with *E. coli* OP50 and treated with 2,7-BCZ (1 μM, dissolved in 0.1% DMSO) at the L1 stage, while control worms received 0.1% DMSO vehicle alone. The 1 μM concentration was selected based on previous studies demonstrating neurobehavioral effects in *C. elegans* at environmentally relevant concentrations.^14^ Experiments were conducted with three biological replicates, each containing 50–100 worms *per* condition, and worms were analyzed at days 2, 4, and 6 posthatching to assess age-dependent α-synuclein aggregation.

α-Synuclein::YFP aggregates were visualized using a confocal microscope equipped with a 488 nm laser for YFP excitation. Worms were immobilized with 10 mM levamisole on 2% agarose pads and imaged at a 40× magnification.

To evaluate the α-synuclein expression level, YFP fluorescence intensity in synchronized NL5901 worms was quantified. At least 40 worms *per* group were randomly selected and imaged. The integrated fluorescence intensity was analyzed by using ImageJ software.

### 2.9 Analysis of Microarray Data

Microarray data were obtained from the following GEO database: GSE20295, GSE42966, GSE20333, GSE20164, and GSE49036. All data sets were derived from postmortem substantia nigra tissues. The sample characteristics are summarized as follows: GSE20295 included 18 control samples (age range: 41–94; 5 females and 13 males) and 11 PD samples (age range: 67–84; five females and six males). GSE42966 consisted of six control samples (age range: 58–85; one female and five males) and nine PD samples (four females and five males; four with Braak stage 3 and five with Braak stage 4). GSE20333 comprised six control samples (age range: 68–88; one female and five males) and six PD samples (age range: 70–87; four females and two males). GSE20164 contained five control samples (age range: 72–90; four females and one male) and six PD samples (age range: 74–87; two females and four males). GSE49036 included eight control samples (age range: 60–81; sex information unavailable) and 16 PD samples (eight with Braak stages 3–4 and eight with Braak stages 5–6; sex information unavailable).

In total, 47 PD samples and 43 control samples were included in the integrated analysis. A systematic pipeline was employed to integrate multibatch gene expression data. Initially, quantile normalization was applied to the raw expression values, followed by cross-data set batch-effect correction using the “removeBatchEffect” function from the R package “limma” to minimize technical biases introduced by nonbiological factors. Data quality was assessed through principal component analysis visualization and quantitative metrics. After correction, samples exhibited adequate mixing, and expression medians converged across samples, demonstrating effective removal of batch effects and confirming that the data quality meets the requirements for subsequent analyses.

Differential gene expression analysis was performed using “limma”, and gene enrichment analysis was conducted as described in Section 2.3. While age and sex were noted as potential confounding factors, their overall distribution across groups was balanced. Nevertheless, these variables could not be fully controlled for in the analysis.

All animal experiments were performed following the protocols evaluated and approved by the Hainan University Animal Welfare and Ethics Committee (Ethics Approval Number: HNUAUCC-2024-00063).

### 2.10 Cell Viability Assay

Cell viability of SH-SY5Y cells was determined using the CCK-8 assay (catalog no. C0038, Beyotime, China). Briefly, cells were seeded in 96-well plates and treated with PHCZs for 24 h, after which 10 μL of CCK-8 solution was added to each well and incubated at 37 °C for 1 h before measuring the absorbance at 450 nm with a microplate reader.

### 2.11 Analysis of Transcriptome Data

SH-SY5Y cells were exposed to 100 μM 2,7-BCZ dissolved in 0.1% DMSO, while control cells received 0.1% DMSO vehicle alone. The concentration of 100 μM in SH-SY5Y cells was selected based on preliminary cytotoxicity assays and literature on PHCZs.^55,56^ Although higher than typical environmental levels,^14^ this dose allows mechanistic exploration of toxicity pathways. Future studies should include lower, environmentally relevant concentrations. Total RNA was extracted from the cells, and the RNA concentration and purity were assessed using a Qubit spectrophotometer (ThermoFisher). Sequencing was performed on the DNBSEQ-T7 platform (BGI). The reference genome and human genome annotation file used was GRCh38.p14 (GCA_000001405.29), obtained from Ensembl (https://www.ensembl.org/Homo_sapiens). RNA-seq was performed with four biological replicates *per* group, yielding an average of 21 million reads *per* sample and normalized using the DESeq2 median-of-ratios method. Differential gene expression analysis was performed using the R package “DESeq2”, with a threshold for identifying differentially expressed genes set at |log2FoldChange| > 1 and *P* < 0.05.

## 3 Results

### 3.1 Physicochemical Properties and Environmental Toxicity Assessment of PHCZs

Traditional toxicology studies on PHCZs typically focus on the representative congeners, overlooking structural variants that may predominate in the environment or exhibit greater toxicity under specific conditions. To address this limitation, we assessed the potential environmental toxicities of 13 PHCZs. Structural formulas and SMILES representations of these 13 PHCZs were retrieved from the PubChem database (Figure 1A). Toxicity predictions conducted using ADMETlab revealed that the LogP values, indicating lipophilicity, did not consistently correlate with an increasing molecular weight (Figure 1B). This observation may be attributed to halogen substituents, with chlorinated derivatives exhibiting the lowest lipophilicity, followed by brominated and then iodinated derivatives. Although the toxicity of PHCZs generally increased with molecular weight, the bioconcentration factors (BCF) for 3-BCZ and 3,6-ICZ were notably lower (Figure 1C).

**Figure 1:**
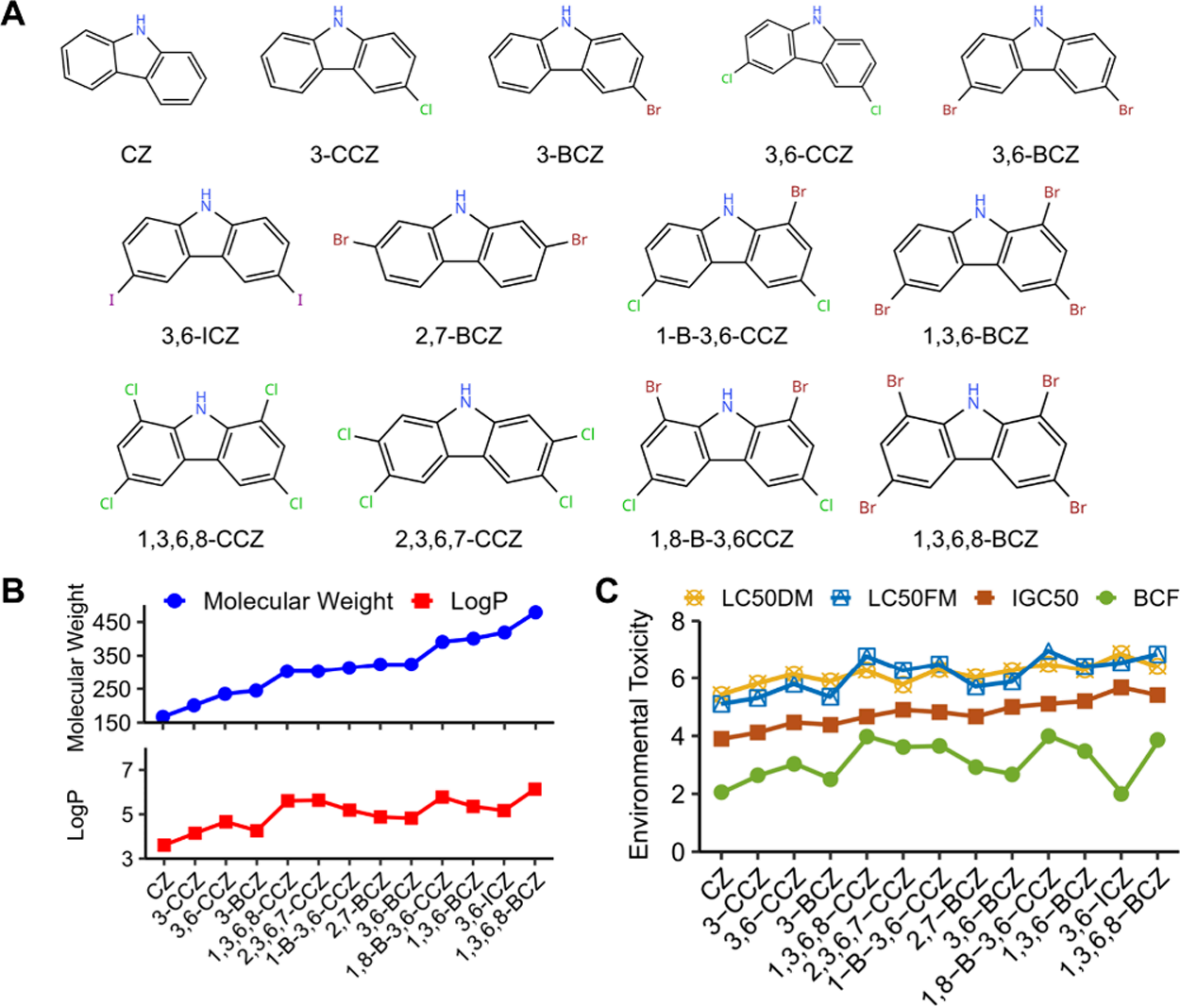
Physiochemical properties and toxicity prediction of PHCZs using ADMETlab. (A) Chemical structures of the 13 PHCZs. (B) Basic properties (molecular weight and LogP as an indicator of lipophilicity) of the 13 PHCZs. (C) Toxicity predictions for the 13 PHCZs, including LC50DM (median lethal concentration after 48 h for the flea *Xenopsylla cheopis*), LC50FM (median lethal concentration after 96 h for the fish*Pimephales promelas*), IGC50 (50% growth inhibition concentration for the beetle*Tenebrio molitor*), and BCF (bioconcentration factor).

### 3.2 Identification of Potential Targets for PHCZs in PD Pathogenesis

To identify potential molecular targets of PHCZs in PD, we retrieved PD-associated genes from GeneCards and OMIM (Tables S1 and S2). Potential PHCZ targets were predicted computationally using SuperPRED, NetInfer, and SEA (Table S3). The intersection of these data sets yielded a core set of 68 candidate targets that possibly mediate PHCZ neurotoxic effects in PD (Figure 2A). Gene enrichment analysis revealed that these overlapping targets were primarily associated with pathways including regulation of membrane potential, distal axon function, postsynaptic neurotransmitter receptor activity, neuroactive ligand–receptor interactions, dopaminergic synapse, and neuroactive ligand signaling (Figure 2B,C).

**Figure 2:**
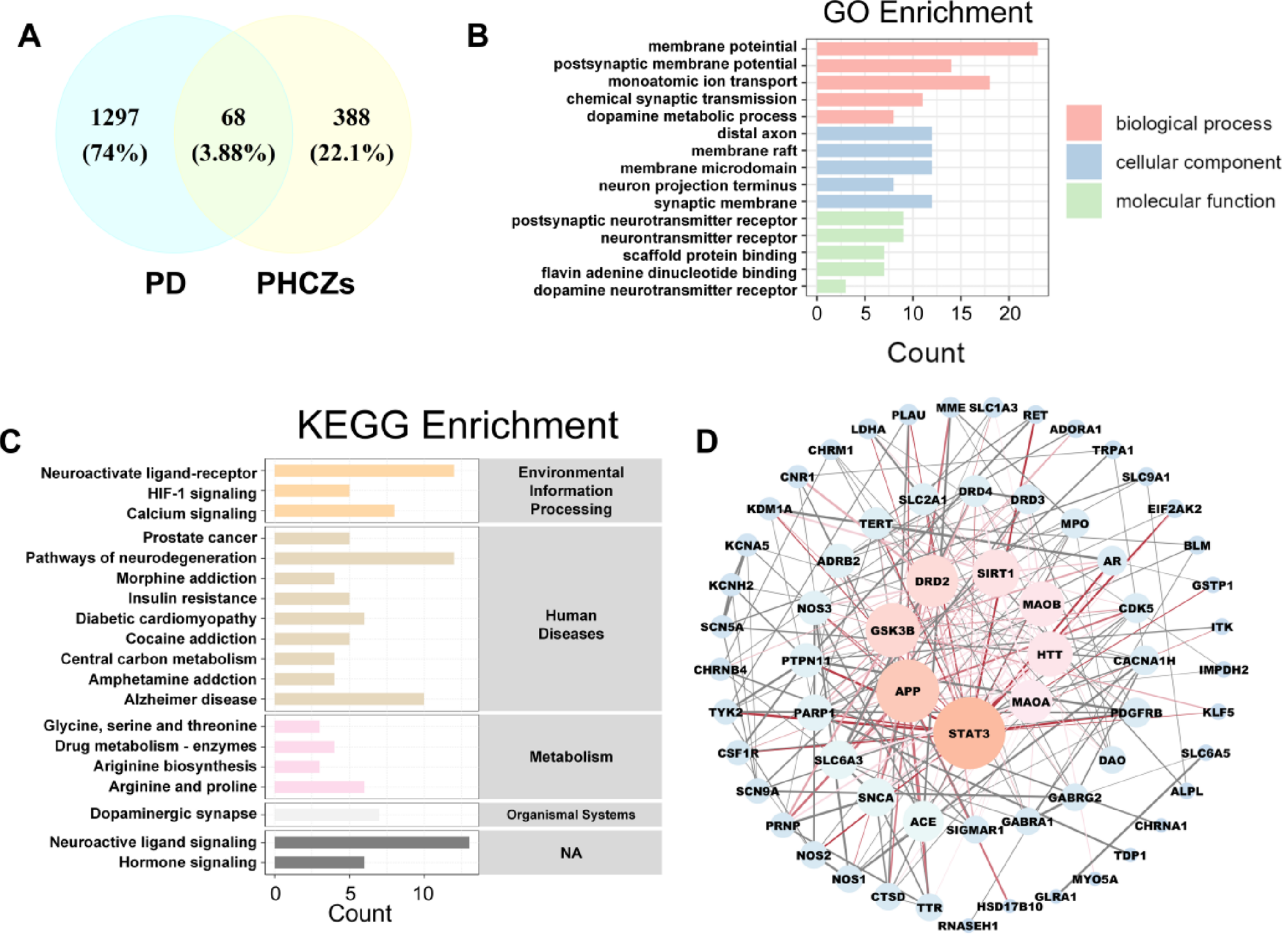
Identification of PHCZ-associated disease targets in PD. (A) *Venn* diagram showing the overlap of PHCZs and PD targets. (B) Gene ontology (GO) enrichment analysis results. (C) Kyoto Encyclopedia of Genes and Genomes (KEGG) enrichment analysis results. (D) Protein–protein interaction (PPI) network analysis results.

We next constructed a protein–protein interaction (PPI) network for the 68 overlapping targets by using data from the STRING database. Within this network, eight hub targets with the highest degree of connectivity were identified: STAT3, APP, GSK3B, DRD2, SIRT1, MAOB, HTT, and MAOA (Figure 2D). These eight genes were thus defined as key hub targets.

### 3.3 Structural Validation of PHCZ Direct Interactions with Core Targets via Molecular Docking

To investigate the binding affinity of PHCZs with the eight hub targets, molecular docking was performed for all of the PHCZs against these proteins. MAOB exhibited the strongest average binding affinity to PHCZs (average vina score = −7.8 kcal/mol, Figure 3A). Although 1-bromo-3,6-dichlorocarbazole (1-B-3,6-CCZ) exhibited the highest binding affinity, we ultimately selected 2,7-BCZ for further analysis, given that dihalogenated PHCZs are generally more common and abundant compared to other homologues (Figure 3B,C).

**Figure 3:**
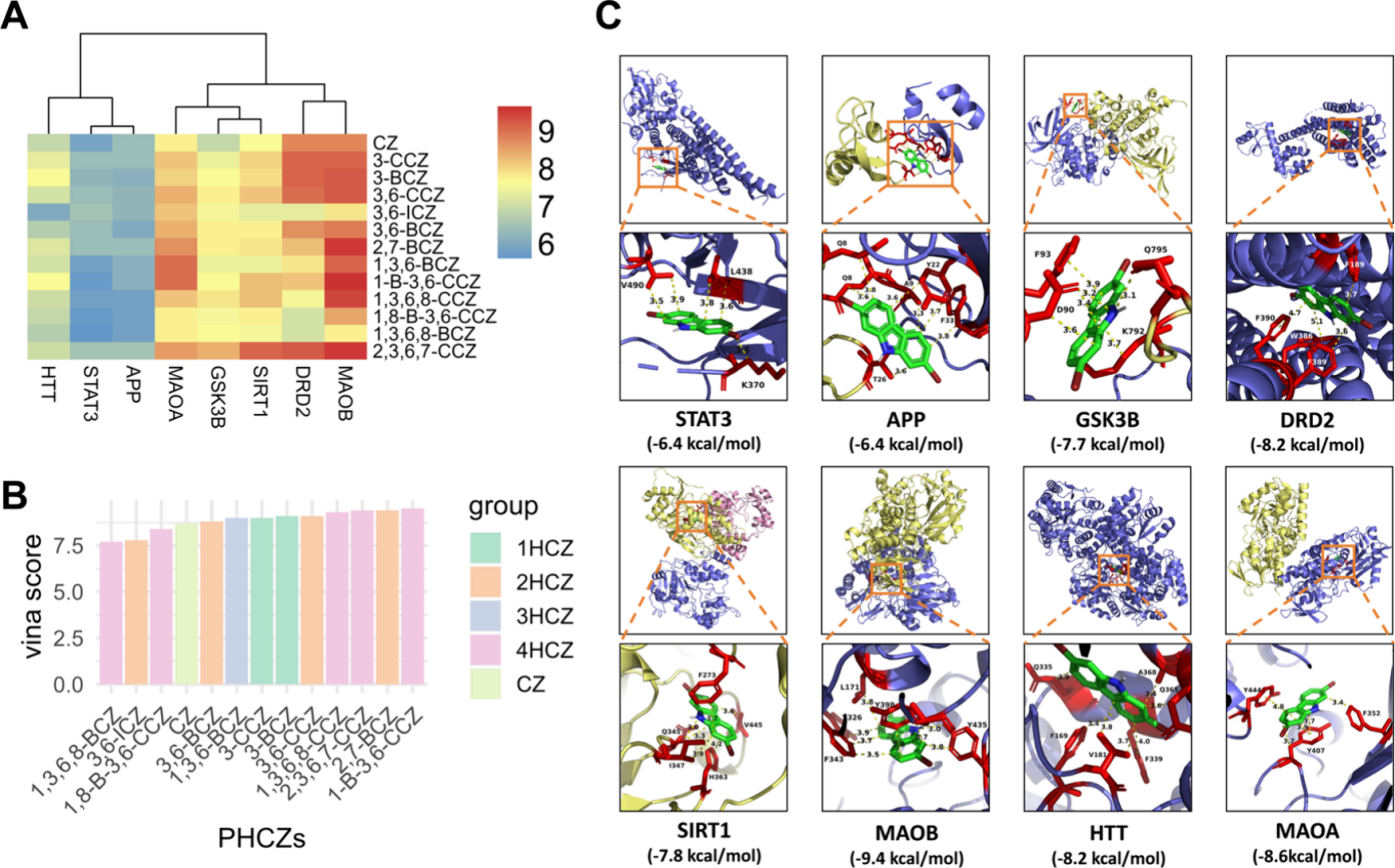
Binding affinity analysis of PHCZs with the eight hub targets via molecular docking. (A) Vina scores (kcal/mol) of 13 PHCZs across eight hub targets. (B) Vina scores (kcal/mol) for MAOB with 13 PHCZs. (C) Representative visualization of docking results between 2,7-BCZ and eight hub targets.

### 3.4 *In* *Vivo* Validation of 2,7-BCZ Highly Correlate with α-Synuclein Aggregation

2,7-BCZ, widely detected in the environment and extensively used as an industrial intermediate in the synthesis of photoelectric materials,^57^ has raised concerns about human exposure and associated adverse effects. Molecular docking simulations indicated that 2,7-BCZ displayed the highest binding affinity for MAOB (−9.4 kcal/mol, an enzyme catalyzing dopamine metabolism) among all tested PHCZs (Figure 3C), suggesting potential interference with dopamine homeostasis.

Furthermore, comparative analysis of the molecular docking results revealed that 2,7-BCZ and the canonical MAOB inhibitor A1IDI occupy an almost identical binding pose within the enzyme’s active site (Figure 4A,B).^58^ This high degree of spatial overlap further corroborates that 2,7-BCZ functions as an MAOB inhibitor, reinforcing the plausibility of its disruptive effects on dopamine metabolism.

**Figure 4:**
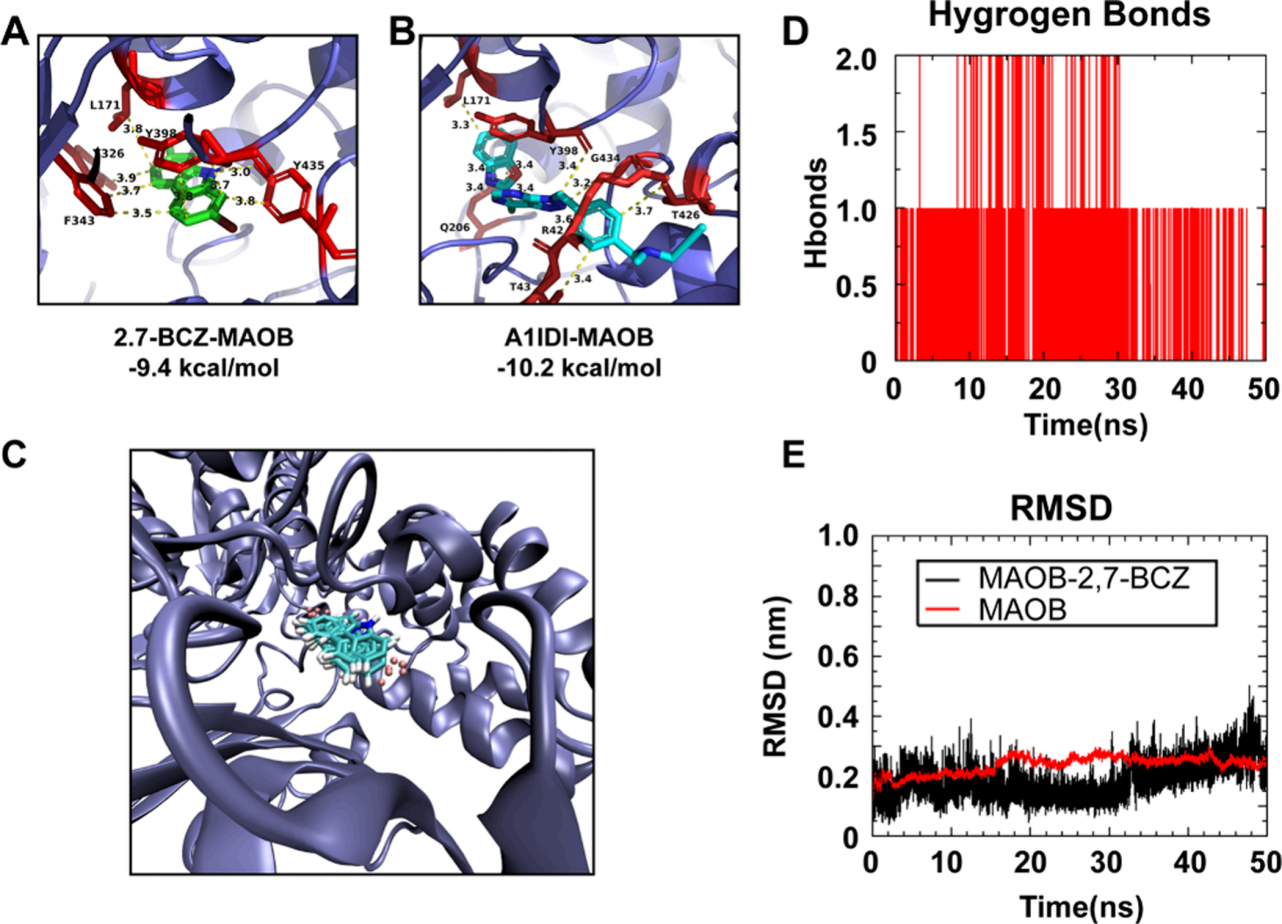
Results of molecular docking and molecular dynamics simulations for 2,7-BCZ with MAOB. (A) Molecular docking diagrams of 2,7-BCZ with MAOB. (B) Molecular docking diagrams of A1IDI with MAOB. (C) Molecular dynamics simulation plot of 2,7-BCZ bound to MAOB. (D) Number of hydrogen bonds observed during the molecular dynamics simulation of 2,7-BCZ with MAOB. (E) Root-mean-square deviation (RMSD) plot from the molecular dynamics simulation of 2,7-BCZ with MAOB.

Further, we performed molecular dynamics simulations on the docking results of MAOB and 2,7-BCZ. The radius of gyration (Rg), root-mean-square fluctuation (RMSF), and solvent accessible surface area (SASA) analyses consistently demonstrate that the MAOB–2,7-BCZ complex is structurally stable over the 50 ns MD simulation (Supporting Information Figures S1–S3). Moreover, MM/PBSA binding free energy calculations based on the MD trajectory yielded a total binding free energy of −35.84 kJ/mol, further corroborating the favorable and stable interaction. The compactness of the protein is preserved, residue-level flexibility remains within acceptable limits, and solvent exposure shows no signs of destabilization. These results suggest that 2,7-BCZ forms a stable complex with MAOB and does not induce unfavorable conformational changes.

Visualization of the binding pose reveals that 2,7-BCZ occupies the catalytic pocket through hydrophobic and polar interactions, supporting its role as a potential inhibitor (Figure 4C). The molecular dynamics simulation demonstrates sustained hydrogen bonding between MAOB and 2,7-BCZ after initial equilibration, indicating stable ligand–protein interactions (Figure 4D). The complex exhibited minimal structural perturbation relative to free MAOB, with root-mean-square deviation (RMSD) values confirming the overall conformational stability throughout the 50 ns trajectory (Figure 4E). Quantitative MAOB assays further indicated that exposure to 2,7-BCZ induced an increase in MAOB protein expression, which may represent a compensatory cellular response resulting from the inhibition of MAOB enzymatic activity by 2,7-BCZ (Figure S4).

While MAOB inhibition is a therapeutic strategy for PD, chronic, unregulated inhibition by this environmental pollutant could disrupt dopaminergic homeostasis, potentially leading to neurotoxic outcomes and contributing to PD pathogenesis. This provides a direct molecular mechanism linking 2,7-BCZ exposure to neurotoxicity relevant to Parkinson’s disease. These considerations lead us to focus on 2,7-BCZ for downstream *in vitro* and *in vivo* neurotoxicity studies.

To assess whether 2,7-BCZ may contribute to PD-like pathology, we exposed the transgenic *C. elegans* strain NL5901 that express human α-synuclein fused to yellow fluorescent protein [*P*_*unc‑*54_::α-synuclein::YFP+*unc-*119] in the body wall muscles, to 1 μM 2,7-BCZ. This strain serves as a well-established model for screening compounds that evaluate PD.^59,60^ Exposure to 1 μM 2,7-BCZ markedly promoted α-synuclein aggregation (Figure 5A). Although the observed aggregation occurs in body wall muscles rather than neurons, this response reflects early protein toxicity and cellular stress responses that are relevant to the broader pathogenesis of PD.

**Figure 5:**
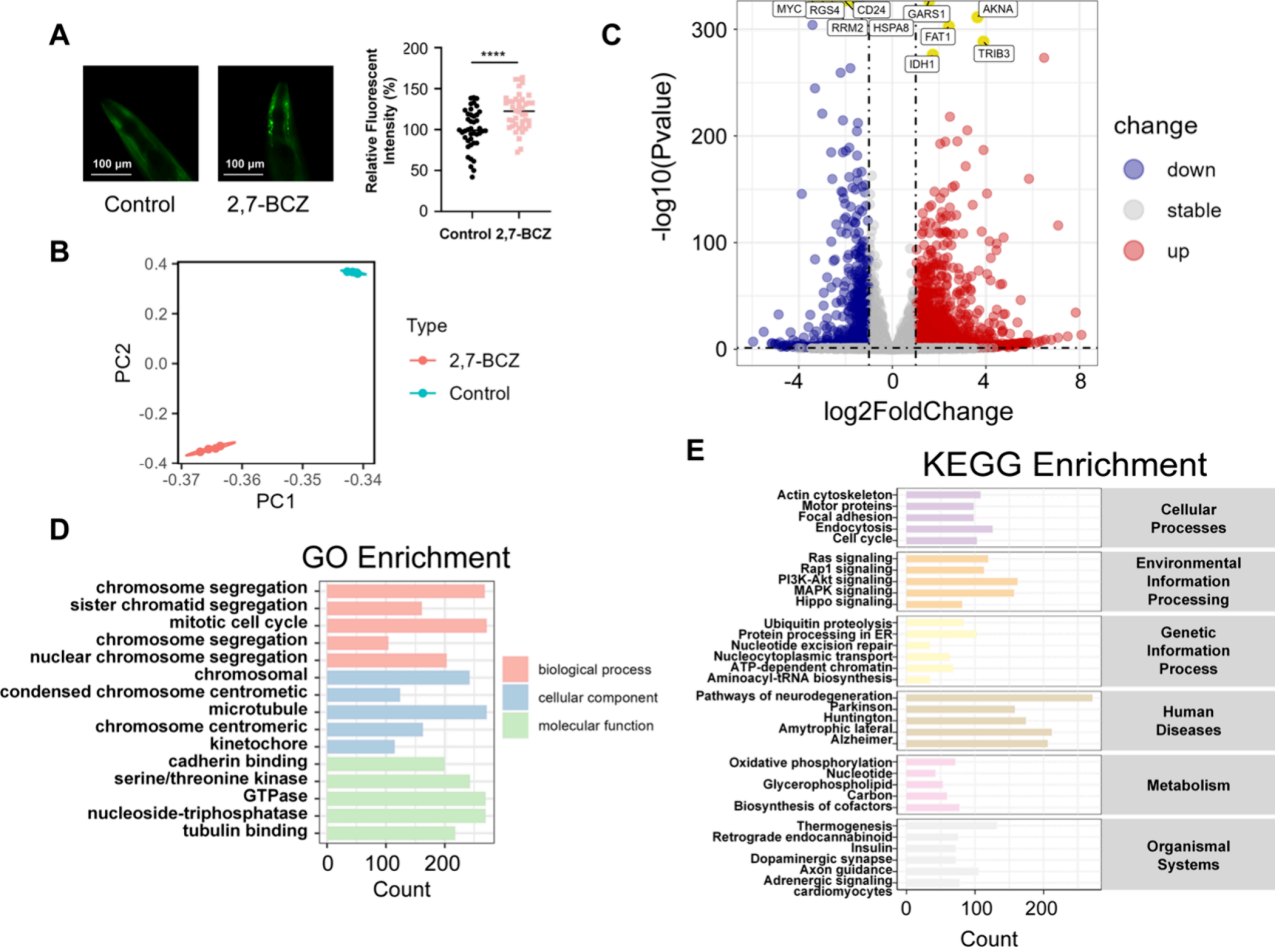
Effects of 2,7-BCZ on α-synuclein aggregation in *C. elegans* and transcriptomic analysis of 2,7-BCZ-treated SH-SY5Y cells. (A) Representative images of α-synuclein aggregation in *C. elegans* body wall muscles. Green fluorescence represents α-synuclein tagged with yellow fluorescent protein specifically expressed in *C. elegans* muscle cells exposed to either DMSO (control) or 1 μM 2,7-BCZ in DMSO solution. (B) Principal component analysis (PCA) of the transcriptome in SH-SY5Y cells exposed to 100 μM 2,7-BCZ. (C) Differentially expressed genes in SH-SY5Y cells exposed to 100 μM 2,7-BCZ. (D) GO enrichment analysis results. (E) KEGG enrichment analysis results. Statistic differences were determined by *t*-test, ****P < 0.0001.

### 3.5 Transcriptomic Characterization of 2,7-BCZ-Treated SH-SY5Y

The 100 μM concentration was selected for transcriptomic profiling to ensure the detection of robust gene expression changes for mechanistic exploration based on preliminary cytotoxicity assays indicating that this dose induced significant biological effects (Figure S5). Although higher than typical environmental levels, this concentration enables a comprehensive investigation into the potential gene regulatory networks perturbed by 2,7-BCZ. Principal component analysis (PCA) revealed substantial whole genome level transcriptomic alterations in SH-SY5Y cells following 2,7-BCZ exposure (Figure 5B). Differential expression analysis identified a total of 9126 significantly dysregulated genes (|log_2_FoldChange| > 1, *P* < 0.05, Table S4 and Figure 5C), indicating that 2,7-BCZ exerts a broad impact on gene regulatory networks in dopaminergic cells, since SH-SY5Y is an immortalized human neuron cell line that has potential to differentiate into dopaminergic neurons. Gene enrichment analysis showed that the differentially expressed genes were primarily involved in the chromosomal region, cadherin binding, regulation of actin cytoskeleton, and neurodegeneration in multiple diseases (Figure 5D,E, *P*_adj_ < 0.05).

### 3.6 Shared Transcriptional Signature of PD and 2,7-BCZ Exposure

To explore the link between 2,7-BCZ and PD, we analyzed substantia nigra microarray data sets from PD patients and healthy donors (controls) in GEO database (GSE20295, GSE42966, GSE20333, GSE20164, and GSE49036), comprising 90 samples in total (47 PD cases and 43 controls) (Tables S5–S7). Batch effects across data sets were corrected using the “removeBatchEffect” function (Figure 6A and Figure S6). Differential expression analysis identified 17 significantly dysregulated genes (|log_2_FoldChange| > 1, *P* < 0.05, Figure 6B and Table S8), which were primarily enriched in pathways such as dopamine biosynthetic process, neuron projection terminus, dopamine binding, PD, tyrosine metabolism, and synaptic vesicle cycle (Figure 6C,D, *P*_adj_ < 0.05). Notably, *CLSTN2*, *CBLN1*, *AGTR1*, *DLK1*, and *DDC* were consistently downregulated in both data sets (log_2_FoldChange < −1), suggesting shared transcriptional perturbations that may underlie the mechanistic link between 2,7-BCZ exposure and PD (Figure 6E).

**Figure 6:**
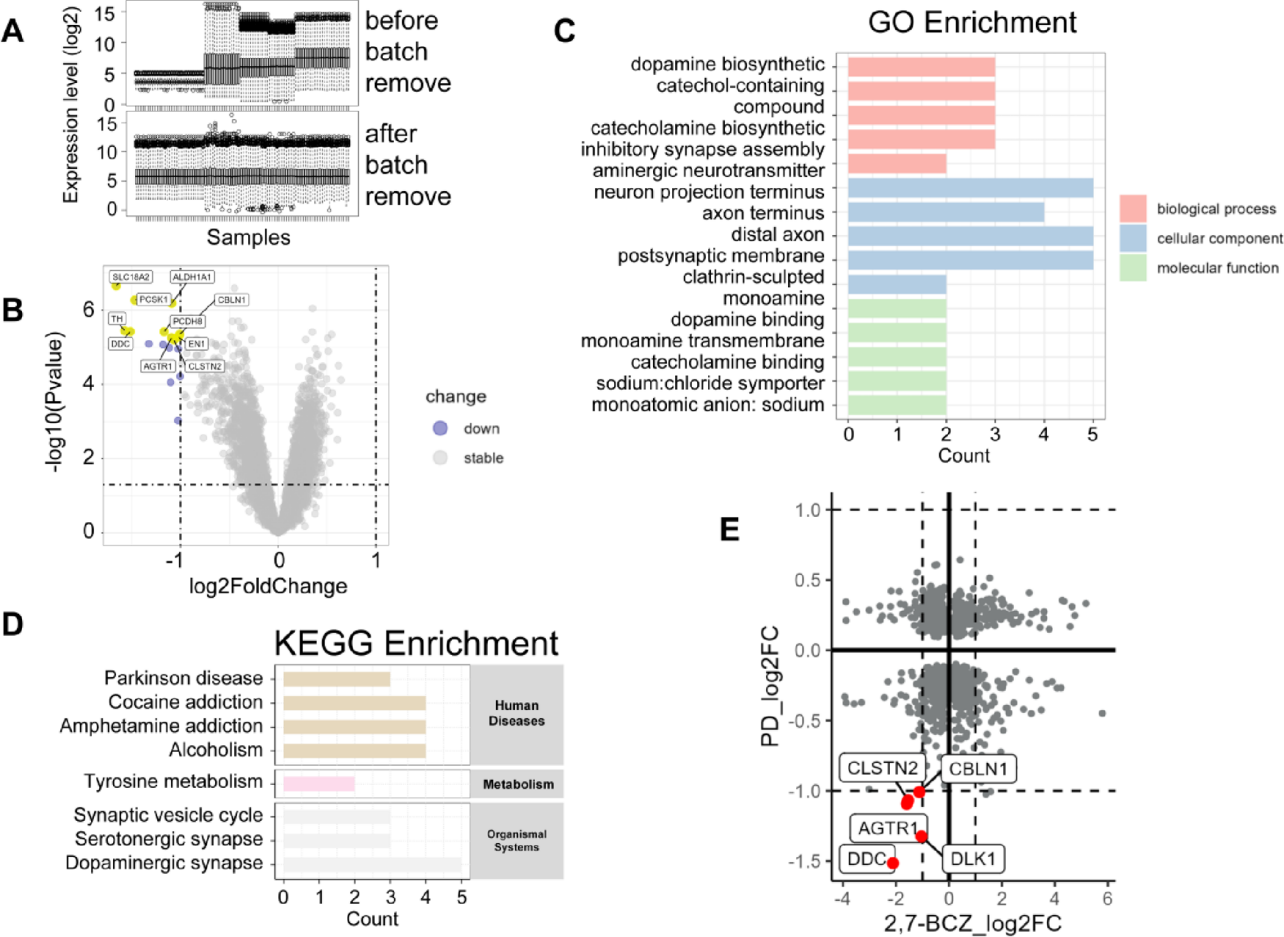
Analysis of PD microarray data from public databases. (A) Distribution of transcriptomic profiles before (left) and after (right) batch-effect correction. (B) Volcano plot illustrating the differentially expressed genes (DEGs) identified from the PD microarray data set. (C) GO enrichment analysis results. (D) KEGG enrichment analysis results. (E) Scatter plot of genes with *P* < 0.05, where the *x*-axis denotes fold change in 2,7-BCZ-treated SH-SY5Y cells versus control, and the *y*-axis denotes fold change in the PD samples versus control. Commonly downregulated genes in both groups are highlighted in red, indicating shared transcriptional perturbations between 2,7-BCZ exposure and PD.

### 3.7 Comprehensive Analysis of the 2,7-BCZ Toxicity Mechanism in PD

To elucidate the pathways through which 2,7-BCZ may contribute to PD, we examined the intersection of three pathway sets: S1 (enriched pathways of overlapping target genes), S2 (enriched pathways of differentially expressed genes in the PD transcriptome), and S3 (enriched pathways of differentially expressed genes in the 2,7-BCZ-exposed transcriptome) (Figure 7A). Intersected pathways included dopaminergic synapse, multicellular organism growth, forebrain development, regulation of nervous system development, amino acid metabolic process, regulation of synapse organization, regulation of synapse structure or activity, regulation of cell size, cerebellum development, and mesencephalon development (Figure 7B). These findings highlight key neurodevelopmental and neurotransmission-related processes that are potentially impaired by 2,7-BCZ in the context of PD.

**Figure 7:**
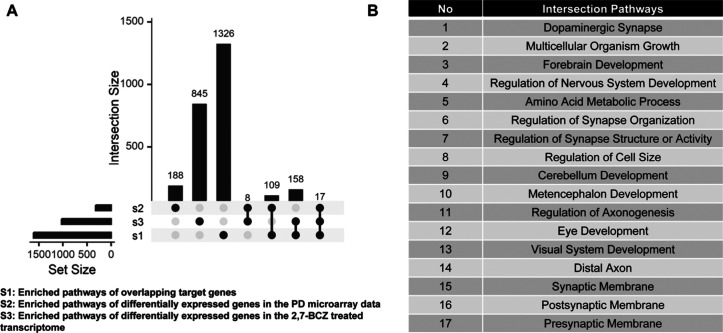
Comprehensive analysis of the toxicity mechanism of 2,7-BCZ. (A) Upset plot illustrated intersections among three pathway sets: S1 (overlapping target gene pathways), S2 (PD transcriptomic pathways), and S3 (2,7-BCZ-treated SH-SY5Y pathways). (B) Table listing intersected pathways across the three sets.

## 4 Discussion

In recent years, NAMs, which integrate computational and experimental approaches, have emerged as a powerful strategy for elucidating the complex toxicity mechanisms of pollutant.^61–63^ The present study was conducted within this framework. We systematically evaluated the PD-related neurotoxicity of PHCZs using a multifaceted approach that integrated toxicity prediction, network toxicology, molecular affinity assessment, *in vivo* validation, and transcriptomic analysis.

Environmental analysis and toxicity predictions revealed that halogen type and molecular weight influence PHCZ lipophilicity and bioaccumulation, consistent with prior reports that brominated carbazoles tend to accumulate in biological systems^5,7,15^ (Figure 1B,C). Network toxicology identified eight hub targets (STAT3, APP, GSK3B, DRD2, SIRT1, MAOB, HTT, and MAOA) shared between PHCZs and PD-associated genes (Figure 2D), emphasizing multiple potential molecular interactions resulting in neurotoxicity. Among these targets, MAOB demonstrated the highest binding affinity with PHCZs (Figure 3B,C). MAOB is a critical enzyme in dopamine metabolism and serves as an important therapeutic target for PD.^64^ Considering both environmental prevalence and binding affinity for MAOB, we selected 2,7-BCZ for further analysis. Comparative analysis with the known MAOB inhibitor A1IDI suggested that 2,7-BCZ likely acts as an MAOB inhibitor (Figure 4A,B),^58^ a conclusion further supported by molecular dynamics simulations (Figure 4C).

*In vivo* experiments using transgenic *C. elegans* overexpressing α-synuclein confirmed that 2,7-BCZ significantly aggravated α-synuclein aggregation, a hallmark of PD pathology (Figure 5A), supporting its role in protein misfolding and neurodegeneration. Transcriptomic profiling of SH-SY5Y cells treated with 100 μM 2,7-BCZ identified 9126 differentially expressed genes (Figure 5C). Enrichment analysis highlighted dopaminergic synapse function, synapse organization, and neurodevelopmental pathways (Figures 5D,E and 6C,D), suggesting that 2,7-BCZ may impair neuronal connectivity and neurotransmission. The observed changes at this relatively high concentration likely represent a composite effect of specific responses and generalized cellular stress. We further emphasize that future studies employing lower, environmentally relevant concentrations combined with transcriptomic analysis will be essential to distinguish specific neurotoxic mechanisms from general cytotoxic effects. Integration with PD patient microarray data sets identified a set of consistently downregulated genes (*CLSTN2*, *CBLN1*, *AGTR1*, *DLK1*, and *DDC*) (Figure 6E), suggesting shared transcriptional perturbations between PHCZ exposure and PD. *DDC* encodes aromatic amino acid decarboxylase, the enzyme that converts L-DOPA to dopamine. Consequently, decreased *DDC* expression suggests a reduction in dopaminergic synthetic capacity required for dopamine replacement with L-DOPA in PD.^65^ Additionally, the downregulation of *AGTR1* has been documented in PD by studies such as that of *Aung et al.* ^66^ In contrast, there is currently no definitive evidence linking *CLSTN2*, *CBLN1*, or *DLK1* to the pathogenesis of PD.

Comprehensive pathway integration of PHCZ target genes, PD transcriptomes, and 2,7-BCZ-treated SH-SY5Y transcriptomes revealed convergent pathways, including dopaminergic synapse regulation, forebrain and cerebellum development, and synapse structural organization (Figure 7B). These findings underscore the potential risk of 2,7-BCZ in disrupting both neurotransmission and neurodevelopmental processes. These adverse consequences also imply that neurons in maturation are more susceptible to the PHCZ’s harmful effect, suggesting a possible intervening time window. In addition to the proposed MAOB-dopamine pathway, PHCZs are known AHR agonists, and AHR activation has been linked to oxidative stress and neuroinflammation, both implicated in PD.^67^ Future studies should explore these complementary mechanisms.

Despite the mentioned discoveries, several limitations of this study should be considered. First, *C. elegans* and SH-SY5Y models, albeit informative, do not fully recapitulate human neurodegenerative processes.^68^ Additionally, experimental concentrations of 2,7-BCZ may exceed typical environmental exposure levels, especially for cell culture treatment, necessitating future studies on chronic low-dose exposures. Notably, the potential coexposure effects of PHCZs with other prevalent environmental contaminants, such as heavy metals known to impair proteostasis and microplastics that serve as carriers for hydrophobic compounds, were not addressed in this study. These coexposures could potentially alter the neurotoxic outcomes observed in our single-contaminant model, either through additive or synergistic mechanisms. Future investigations employing mixture toxicity designs are warranted to better simulate real-world exposure scenarios and refine neurotoxic risk assessments for PHCZs.

This study demonstrates that 2,7-BCZ, a prevalent PHCZ, promotes α-synuclein aggregation, disrupts dopaminergic signaling and alters neurodevelopmental gene expression, implicating its hazardous role in PD pathogenesis. Integrative analyses of molecular docking, transcriptomics, and patient-derived data sets reveal convergent pathways and shared dysregulated genes, with *in vivo* validation in *C. elegans*, providing mechanistic insight into PHCZs’ neurotoxicity. These findings, generated using animal-sparing NAMs, emphasize the need for environmental monitoring of emerging PHCZ pollutants and underscore the value of multiomics approaches for assessing their neurological risks. Our study supports a model wherein 2,7-BCZ binds to MAOB, potentially disrupting dopamine metabolism, promoting α-synuclein aggregation, and leading to transcriptional dysregulation in key neural pathways.

## Supplementary Material



















## Data Availability

The transcriptomic data set generated in this study has been deposited in the NGDC database under accession number HRA013639.

## Notes

Supplementary figures S1–S6 with corresponding legends (PDF)
Genes associated with Parkinson’s disease obtained from the GeneCards database (Table S1) (XLSX)
Genes associated with Parkinson’s disease obtained from the OMIM database (Table S2) (XLSX)
Predicted PHCZ targets from SuperPRED, NetInfer, and SEA (Table S3) (XLSX)
Differentially expressed genes in 2,7-BCZ-treated SH-SY5Y cells (Table S4) (XLSX)
Metadata of microarray data sets from the GEO database (Table S5) (XLSX)
Grouping information on microarray data sets from the GEO database (Table S6) (XLSX)
Expression data of microarray data sets from the GEO database (Table S7) (XLSX)
Differentially expressed genes in Parkinson’s disease microarray data (Table S8) (XLSX)

## ABBREVIATIONS

PHCZs: polyhalogenated carbazoles
MAOB: monoamine oxidase B
NAMs: New Approach Methodologies
DLCs: dioxin-like compounds
AHR: aryl hydrocarbon receptor
3,6-CCZ: 3,6-dichlorocarbazole
3-CCZ: 3-monochloracarbazole
3-BCZ: 3-monobromocarbazole
PD: Parkinson’s disease
2,7-BCZ: 2,7-dibromocarbazole
ROS: reactive oxygen species
GO: gene ontology
KEGG: kyoto encyclopedia of genes and genomes
PPI: protein–protein interaction
PDB: protein data bank
YFP: yellow fluorescent protein
NGM: nematode growth medium
CZ: carbazole
3,6-ICZ: 3,6-diiodocarbazole
3,6-BCZ: 3,6-dibromocarbazole
1-B-3,6-CCZ: 1-bromo-3,6-dichlorocarbazole
1,3,6-BCZ: 1,3,6-tribromocarbazole
1,3,6,8-CCZ: 1,3,6,8-tetrachlorocarbazole
2,3,6,7-CCZ: 2,3,6,7-tetrachlorocarbazole
1,8-B-3,6-CCZ: 1,8-dibromo-3,6-dichlorocarbazole
1,3,6,8-BCZ: 1,3,6,8-tetracbromocarbazole
LC50DM: median lethal concentration after 48 h for the flea *Xenopsylla cheopis*
LC50FM: median lethal concentration after 96 h for the fish *Pimephales promelas*
IGC50: 50% growth inhibition concentration for the beetle *Tenebrio molitor*
BCF: bioconcentration factor
PCA: principal component analysis
