# Supplementary material for: Integrated New Approach Methodologies Reveal the Potential Role of 2,7-Dibromocarbazole in Parkinson’s Disease via Monoamine Oxidase B Inhibition and Dopaminergic Dysfunction
Source: Environ Sci Technol. 2026 Feb 23;60(9):7159–70. doi: 10.1021/acs.est.5c14167 (PMC12980844; doi:10.1021/acs.est.5c14167)
Supplement: Supplementary file 1 [file es5c14167_si_001.pdf]

Integrated New Approach Methodologies Reveal the Potential Role of 2,7-Dibromocarbazole in Parkinson's Disease via Monoamine Oxidase B Inhibition and Dopaminergic Dysfunction

Xinhe Lu [1,2#], Yuhang Luo [1#], Pei Peng [1], Xingyue Xing [3], Wei Xia [2], Hongyan Yin [4], Hanzeng Li [1], and Shunqing Xu [1*]*

[1]*School of Environmental and Science and Engineering, Hainan University, Haikou 570228, China.*

[2]*Key Laboratory of Environment and Health (HUST), Ministry of Education & Ministry of Environmental Protection, School of Public Health, Tongji Medical College, Huazhong University of Science and Technology, Wuhan 430030, Hubei, China.*

[3] *School of Life and Health Sciences, Hainan University, Haikou 570228, China.*

[4] *School of Tropical Agriculture and Forestry, Hainan University, Haikou 570228, China.*

[#] These authors contributed equally;

[*]Corresponding authors: hanzeng.li@hainanu.edu.cn (Hanzeng Li), xus@hainanu.edu.cn (Shunqing Xu)

Pages:4

Table of contents:

Figure S1 – Comparative PCA plots of microarray data pre- and post-batch effect correction

Figure S2 – Root mean square fluctuation (RMSF) of MAOB from the Molecular Dynamics Simulations of 2,7-BCZ and MAOB

Figure S3 – Results of the Solvent Accessible Surface Area (SASA) from Molecular Dynamics Simulations of 2,7-BCZ and MAOB

Figure S4 – MAOB protein levels in SH-SY5Y cells following exposure to 2,7-BCZ

Figure S5 – Effects of varying concentrations of 2,7-BCZ and 0.1% DMSO on cell

viability

Figure S6 – Comparative PCA plots of microarray data pre- and post-batch effect correction

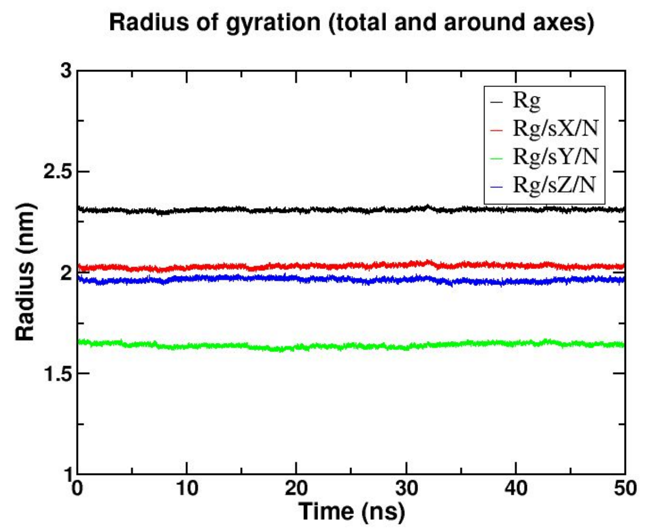

Figure S1. Radius of gyration (Rg) of the MAOB backbone from the molecular dynamics simulations of 2,7-BCZ and MAOB.

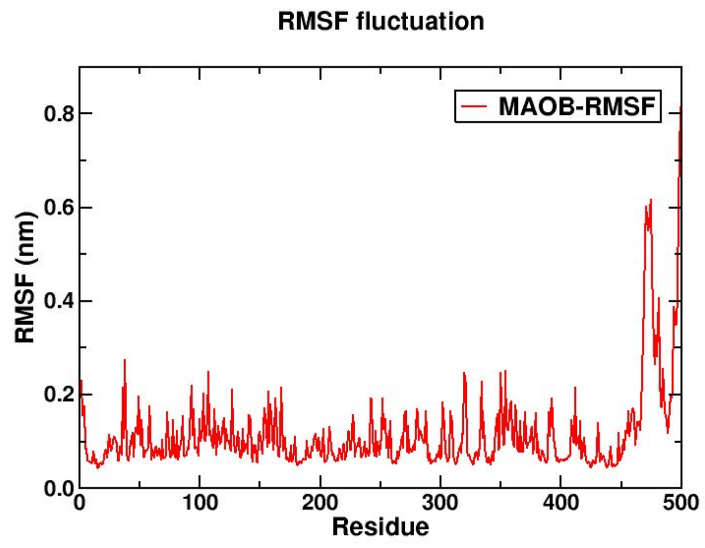

Figure S2. Root mean square fluctuation (RMSF) of MAOB from the Molecular Dynamics Simulations of 2,7-BCZ and MAOB.

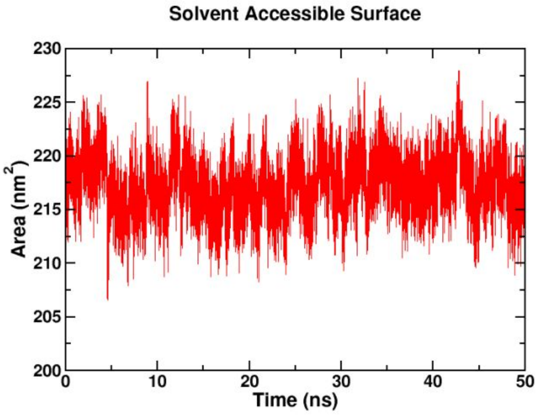

Figure S3. Results of the Solvent Accessible Surface Area (SASA) from Molecular Dynamics Simulations of 2,7-BCZ and MAOB.

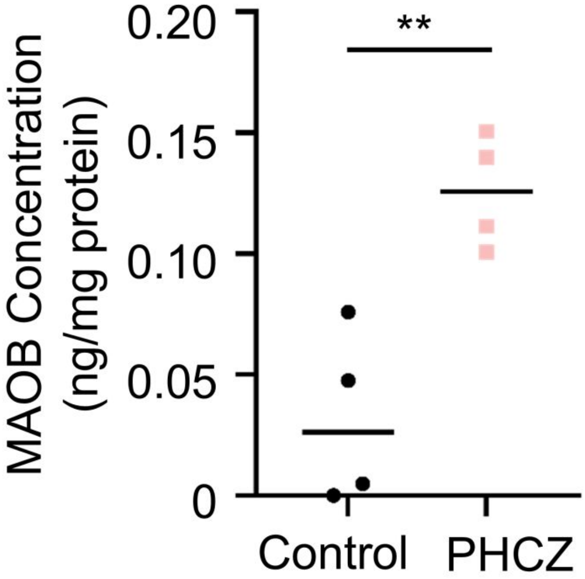

Figure S4. MAOB protein levels in SH-SY5Y cells following exposure to 2,7-BCZ. Statistic differences were determined by *t*-test, and ns represent not significantly different, while ** represent p<0.01.

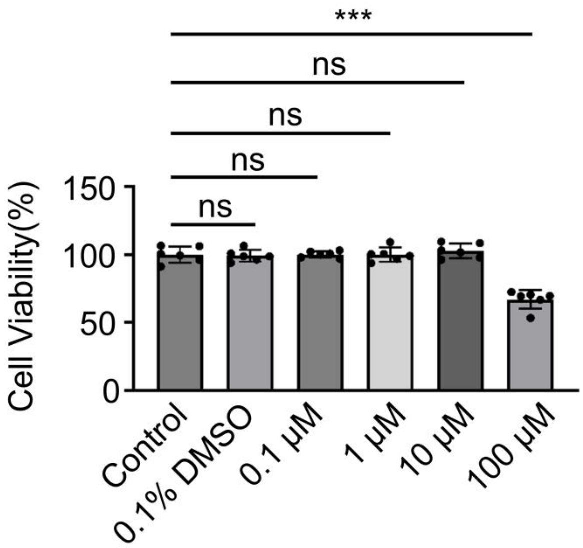

Figure S5. Effects of varying concentrations of 2,7-BCZ and 0.1% DMSO on cell viability. Statistic differences were determined by *t*-test, and ns represent not significantly different, while *** represent $p<0.01$.

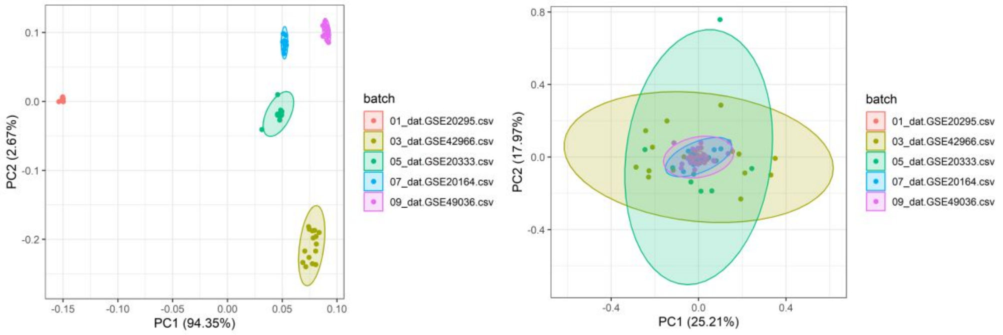

Figure S6. Comparative PCA plots of microarray data pre- and post-batch effect correction.
